# A novel analysis workflow for simultaneous parsing prokaryotic and eukaryotic microbial genes from metagenomes

**DOI:** 10.7717/peerj.20769

**Published:** 2026-02-11

**Authors:** Wei Zhang, Yanmei Zheng, Guomin Han, Xingbing He

**Affiliations:** 1 Anhui Province Key Laboratory of Rice Genetics and Breeding (Rice Research Institute, Anhui Academy of Agricultural Sciences), Hefei, Anhui, China; 2 School of Life Sciences, Anhui Agricultural University, Hefei, Anhui, China; 3 National Engineering Laboratory of Crop Stress Resistance Breeding, Anhui Agricultural University, Hefei, Anhui, China; 4 College of Biology and Environmental Sciences, Jishou Uninversity, Jishou, Hunan, China

**Keywords:** Metagenome, Eukaryotic, Prokaryotic, Gene prediction, Novel workflow

## Abstract

Accurately predicting coding genes from metagenomic samples containing a high proportion of eukaryotic content remains a significant challenge. Novel and reliable methods for the simultaneous prediction of prokaryotic and eukaryotic microbial genes are crucial to address this. We evaluated gene prediction accuracy of MetaGeneMark and MetaEuk using representative genomes from diverse organisms. Based on these findings, we developed an innovative analytical workflow. This approach involves an initial prediction of eukaryotic genes using MetaEuk, followed by the masking of these predicted eukaryotic genes and any co-identified partial prokaryotic genes using a custom Perl script. Remaining prokaryotic genes are then predicted from the masked metagenome using MetaGeneMark or metaProdigal. This integrated strategy achieved similar quantities and average lengths of eukaryotic genes compared to using MetaEuk alone. Notably, the quantity of predicted prokaryotic genes and viral genes using the new workflow was 14–18% higher than that obtained with standalone prokaryotic predictors. Furthermore, validation on a mixed prokaryotic-eukaryotic metagenome demonstrated that our workflow yielded genes with significantly higher average lengths, indicating reduced fragmentation and improved gene integrity. This novel workflow effectively enables the rapid and comprehensive retrieval of high-quality prokaryotic and eukaryotic coding sequences from diverse metagenomes.

## Introduction

The advent of metagenomics, first conceptualized by Handelsman et al. (1998), offered a novel paradigm for studying the collective genomes and biosynthetic machinery of microbial communities, initially focusing on soil microflora. Soil ecosystems harbor a vast diversity of microorganisms, encompassing eubacteria, archaea, and eukaryotes—collectively termed the microbiota—which are integral to processes such as plant growth (Srivastava et al., 2023). Analytical techniques like marker gene surveys, as well as genomic and metagenomic analyses, have been instrumental in characterizing soil microbiomes and their influencing factors (Dubey et al., 2020; Knight et al., 2018). Notably, metagenomic sequencing technology has revolutionized the detection and characterization of microbial species (Lataretu et al., 2025), spurring the development of numerous software tools for the taxonomic classification from these complex datasets (Ye et al., 2019).

Historically, many widely adopted metagenomic analysis pipelines have primarily concentrated on prokaryotic microorganisms, often leading to an under-representation of eukaryotic components (Baehren et al., 2023; Dubay et al., 2023). This focus likely stemmed from the numerical dominance of bacteria in well-studied environments like the human gut and many soil habitats. However, eukaryotic microorganisms are also abundant, particularly in surface soils, where they play crucial roles in nutrient cycling and overall topsoil ecosystem function (Wu et al., 2022). Consequently, the analysis of eukaryotic microbial information within metagenomic sequences is essential for a comprehensive understanding of their ecological roles (Köninger et al., 2023). Encouragingly, software packages capable of analyzing eukaryotic diversity are emerging (Garfias-Gallegos et al., 2022; Liu et al., 2021). In the realm of gene prediction from metagenomes, existing tools are typically specialized for either prokaryotic or eukaryotic gene identification. For instance, MetaGeneMark and metaProdigal are widely utilized for predicting prokaryotic genes (Hyatt et al., 2012; Zhu, Lomsadze & Borodovsky Nar, 2010), whereas MetaEuk is designed for mining eukaryotic microbial protein-coding genes from metagenomes (Levy Karin, Mirdita & Söding, 2020). A significant limitation, however, is that these tools generally do not accurately predict genes across both domains within a single analysis. While EukRep attempts to differentiate eukaryotic and prokaryotic contigs in metagenomics (West et al., 2018), its utility can be constrained by issues such as the misassembly of chimeric eukaryotic-prokaryotic contigs (Pronk & Medema, 2022).

To address the challenge of comprehensive gene prediction across domains, we have developed an integrated workflow capable of simultaneously identifying both prokaryotic and eukaryotic genes from metagenomic data. Our approach involved an initial evaluation of MetaGeneMark and MetaEuk for gene prediction accuracy across diverse organismal types. Based on these findings, we constructed a novel analytical workflow. This workflow prioritizes the prediction of eukaryotic genes using MetaEuk. Subsequently, these predicted eukaryotic genes, along with any partially predicted prokaryotic genes identified by MetaEuk, are masked within the metagenome. The remaining, unannotated sequences in the masked metagenome are then processed using MetaGeneMark or metaProdigal to predict the remaining prokaryotic genes. This staged, integrated strategy enables a more exhaustive recovery of both prokaryotic and eukaryotic microbial coding sequences from complex metagenomic datasets.

## Methods

To assess the gene prediction accuracy of the prokaryotic pipeline MetaGeneMark and the eukaryotic gene prediction pipeline MetaEuk, we analyzed diverse single-species genomes. For this evaluation, five representative genomes and their corresponding annotated protein sequences were selected for each of the following superkingdoms/kingdoms: Archaea, Bacteria, Fungi, Metazoa, and Plant, all downloaded from NCBI (Table 1).

**Table 1:** Genome and protein sequence information of each species.

**Superkingdom**	**Species**	**Number of proteins**
Archae	*Anaerobic archaeon*	3,944
	*Haloterrigena turkmenica*	5,113
	*Methanosarcina horonobensis*	4,095
	*Natrialba magadii*	4,204
	*Salinigranum rubrum*	4,474
Bacteria	*Acinetobacter baumannii*	3,657
	*Bacillus thuringiensis*	6,508
	*Escherichia coli*	5,093
	*Vibrio xuii*	7,324
	*Yersinia pseudotuberculosis*	4,340
Fungi	*Armillaria gallica*	25,695
	*Fusarium graminearum*	14,145
	*Penicillium antarcticum*	11,514
	*Trametes versicolor*	14,296
	*Trichoderma asperellum*	12,557
Metazoa	*Helobdella robusta*	23,432
	*Lottia gigantea*	23,340
	*Onchocerca volvulus*	14,628
	*Pristionchus pacificus*	29,202
	*Zootermopsis nevadensis*	14,610
Plant	*Arabidopsis thaliana*	48,322
	*Brassica rapa*	41,025
	*Glycine max*	88,412
	*Oryza sativa*	42,411
	*Zea mays*	131,585

**Notes.**

The table summarizes protein sequence data for 25 species across five superkingdoms (Archaea, Bacteria, Fungi, Metazoa, and Plant), with five representative species per superkingdom. Protein counts reflect annotated sequences for each species.

**Figure 1:**
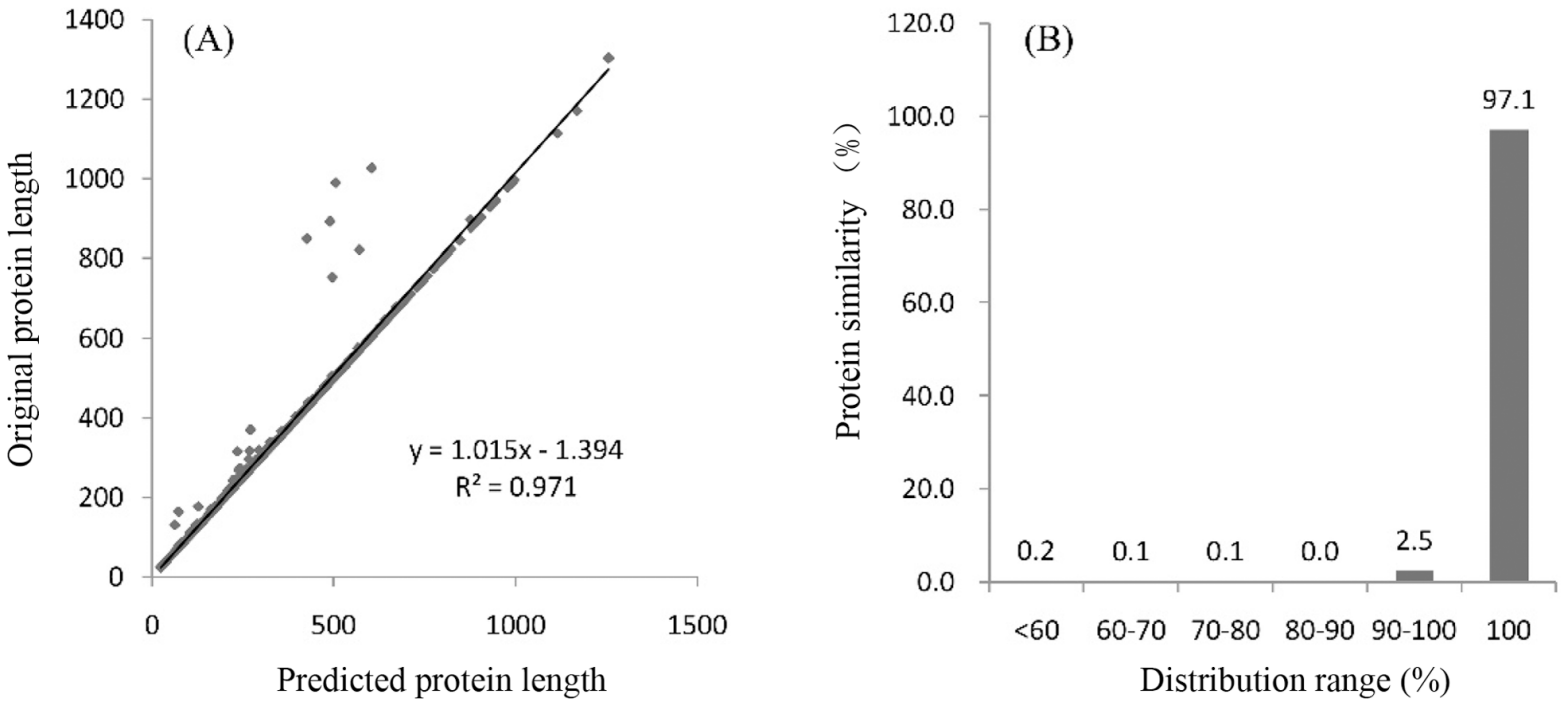
Evaluation results of MetaEuk for prokaryotic organisms. (A) A comparison of the gene length; (B) a comparison of the protein similarly. Note: for a small number of prokaryotic genes predicted by MetaEuk in the prokaryotic prediction results, represented by the results of an archaea, eggNOG was used to evaluate the results. It can be seen from the figure that the prokaryotic results predicted by MetaEuk are very accurate, with 97.1% similarity to the reference sequence proteins.

Based on the evaluation results, we developed a novel analytical workflow for the simultaneous prediction of prokaryotic and eukaryotic genes from metagenomic data (Fig. 1). The workflow proceeds as follows: (1) the assembled metagenomic sequence is first processed with MetaEuk. This step primarily aims to identify eukaryotic organism genes, though some prokaryotic genes may also be detected. (2) Subsequently, a custom Perl script is used to mask the genomic regions corresponding to all genes predicted in this initial step by replacing their sequences with ‘N’ characters (unknown bases). (3) The resulting masked sequences are then subjected to a second round of gene prediction using either MetaGeneMark or metaProdigal to identify remaining prokaryotic genes. (4) Finally, the gene sets predicted by MetaEuk (first pass) and MetaGeneMark/metaProdigal (second pass) are combined to produce a comprehensive set of prokaryotic and eukaryotic gene predictions from the metagenome.

Detailed instructions for implementing this workflow are available on GitHub (https://github.com/wzhangahas/PPEMS). For evaluation, we utilized the benchmark metagenomic dataset from the Tara Oceans project, obtained from the MetaEuk repository (https://wwwuser.gwdguser.de/ compbiol/metaeuk/). This dataset consists of assembled contigs from marine environments, rich in eukaryotic sequences, and served to test our workflow’s performance on complex, eukaryotic-predominant metagenomes. This eukaryote-rich dataset provides a stringent test for eukaryotic gene prediction component of our workflow. The final consolidated set of predicted coding sequences was subjected to functional annotation and orthology assignment using EggNOG-mapper (version 5.0).

In addition, we further validated our workflow using the metagenomic dataset provided by DeepMicroClass (Hou et al., 2024), which contains mixed prokaryotic and eukaryotic contigs. The dataset was assembled by MEGAHIT (v1.2.9). The assembled contigs were analyzed using our integrated workflow, as well as standalone MetaEuk and Metagenemark predictors, followed by functional annotation using EggNOG-mapper.

## Results

### Comparative evaluation of MetaGeneMark and MetaEuk for gene prediction in diverse single species genomes

We utilized MetaGeneMark and MetaEuk to predict genes from the genomes of diverse single species. The results showed that MetaGeneMark successfully predicted both the number and average length of genes for prokaryotes, closely matching the values obtained from the original genome annotations (Fig. S1). Conversely, according to the results obtained from MetaGeneMark when applied to eukaryotic genomes, eukaryotic genes were frequently fragmented, resulting in a higher number of genes and shorter average gene lengths compared to the original values (Fig. S1). These results indicate that MetaGeneMark tends to predict a significantly higher number of spurious or fragmented gene segments in eukaryotes, rendering it unsuitable for eukaryotic gene prediction. It is, therefore, primarily optimized for prokaryotic gene prediction.

The MetaEuk software utilizes a homology-based approach to predict coding genes from genomes. MetaEuk predicted a similar number of eukaryotic genes and achieved comparable average gene lengths to the original values (Fig. S1). These findings suggest that MetaEuk is better suited for eukaryotic organisms and provides more reliable gene models.

**Figure 2:**
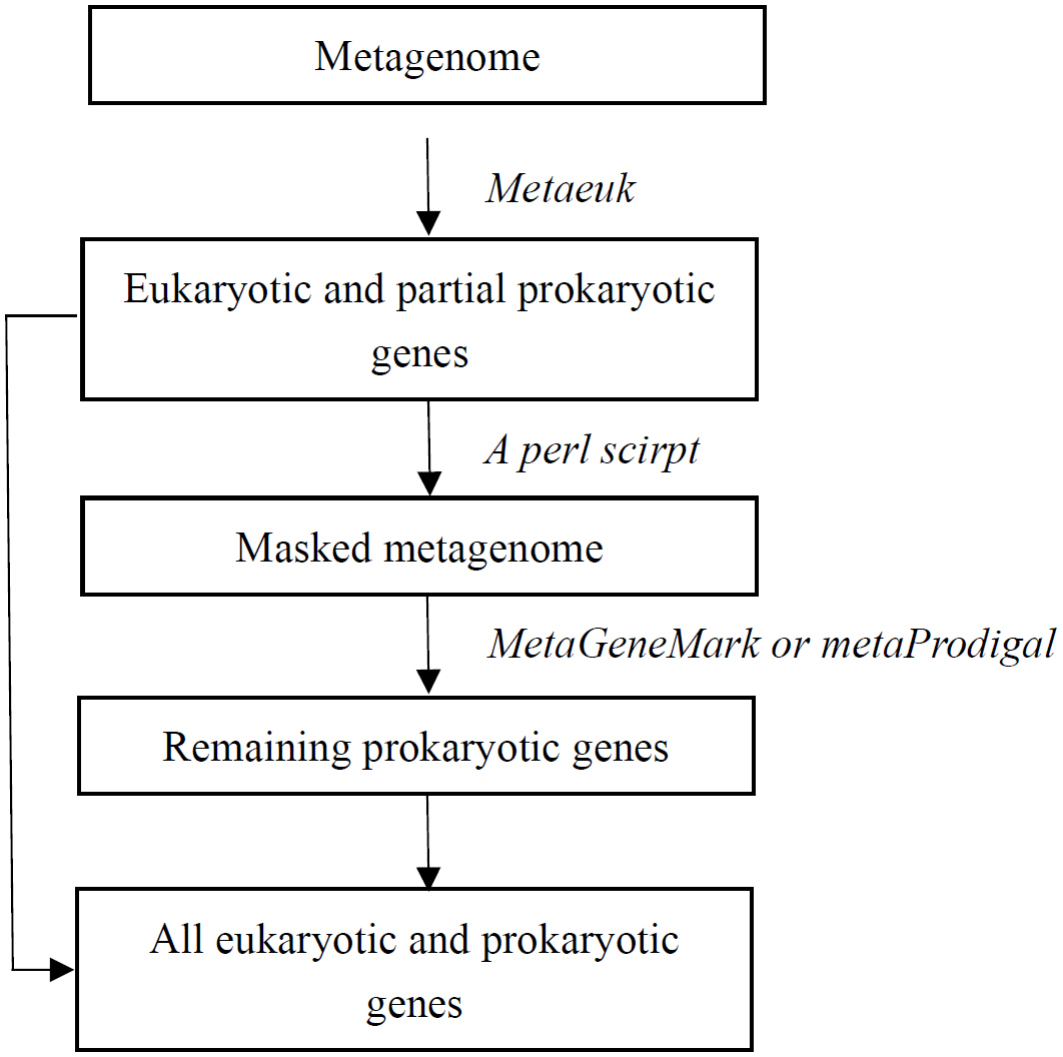
Workflow for simultaneously mining prokaryotic and eukaryotic microbial genes. Note: A workflow that can simultaneously analyse prokaryotic microorganisms, viruses and eukaryotic microorganisms. The workflow involves initially predicting eukaryotic genes using MetaEuk, followed by masking of predicted eukaryotic and partial prokaryotic genes *via* Custom Perl script. The subsequent prediction of remaining prokaryotic genes is conducted using MetaGeneMark or MetaProdigal.

The MetaEuk software is also capable of predicting a subset of prokaryotic genes. However, the predicted number of prokaryotic genes was significantly lower compared to the original gene count, accounting for only approximately 25% of the original count. Nevertheless, the average lengths of the predicted prokaryotic genes were very close to the original values. Through homology-based comparisons, it was determined that the predictions made by MetaEuk for prokaryotic organisms are highly accurate, with an average sequence identity of 97.1% to reference protein sequences (Fig. 2). Therefore, while MetaEuk primarily focuses on predicting eukaryotic genes in metagenomic data, the subset of prokaryotic genes it predicts is relatively accurate. Although the predicted gene count is significantly lower, these predicted prokaryotic coding genes can be directly used for subsequent analysis.

### A workflow for simultaneously mining prokaryotic and eukaryotic microbial genes

Given that MetaEuk is proficient at predicting eukaryotic genes and a minor subset of prokaryotic genes, whereas MetaGeneMark is optimized for prokaryotic gene prediction and performs poorly on eukaryotic sequences (often leading to their fragmentation), a strategic order of application is crucial. Initiating gene prediction with MetaGeneMark would necessitate subsequent complex and computationally intensive steps to distinguish fragmented eukaryotic sequences from true prokaryotic genes. Conversely, employing MetaEuk first yields reliable predictions for the majority of eukaryotic genes and a small, accurate subset of prokaryotic genes, suitable for direct use. Therefore, our novel analytical workflow (Fig. 1) was designed as follows: (1) MetaEuk is used to predict eukaryotic coding genes and a limited number of prokaryotic coding genes from the metagenomic data. (2) These initially predicted genes are then masked. (3) Prokaryotic gene prediction software, such as MetaGeneMark or metaProdigal, is subsequently applied to the masked metagenomic data to identify the remaining prokaryotic coding genes.

A key component of our workflow is an efficient masking step. After MetaEuk identifies potential eukaryotic and a small subset of prokaryotic genes, these sequences must be masked to prevent reprediction by subsequent prokaryotic gene finders. Our initial attempts to use RepeatMasker for this purpose proved impractically slow for large metagenomic datasets; masking an approximately 1 GB dataset took several days, and a 10 GB dataset failed to complete within a month. Recognizing that MetaEuk’s output files contain precise gene location information, we developed a custom Perl script. This script directly parses these locations and replaces the corresponding coding sequences in the metagenomic dataset with ‘N’ characters. This targeted, alignment-free approach reduces the masking time from days or weeks to minutes or hours, significantly improving scalability. Finally, the remaining prokaryotic genes are predicted from this efficiently masked metagenomic sequence using MetaGeneMark or metaProdigal.

The gene predictions from MetaEuk (first pass) and MetaGeneMark/metaProdigal (second pass on masked data) are then consolidated. This final set of predicted coding sequences, encompassing both prokaryotes and eukaryotes, can be subjected to further functional annotation using tools like EggNOG to obtain diverse information such as species origin, Gene Ontology (GO) terms, Kyoto Encyclopedia of Genes and Genomes (KEGG) pathways, Clusters of Orthologous Groups of proteins (COG) categories, gene names, and orthologous group assignments.

**Figure 3:**
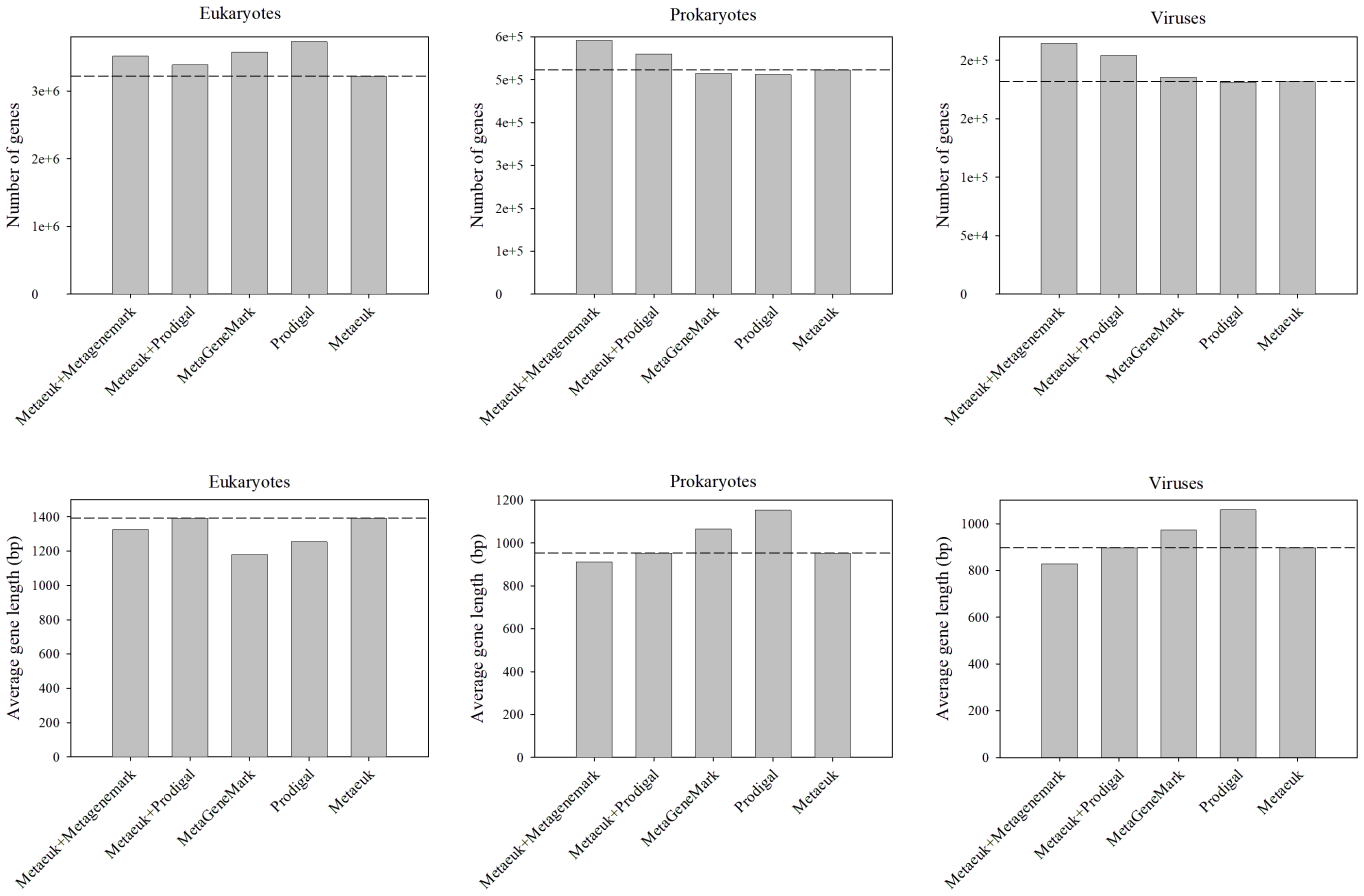
Comparing the new workflow with standalone prokaryotic gene prediction software using real data. To examine this workflow, we analyzed the data in MetaEuk software using a number of different analytical methods, including MetaEuk, MetaGeneMark, and Prodigal. The results showed the number and length of genes calculated by different analysis strategies.

To evaluate our workflow, we utilized a metagenomic dataset known for its high eukaryotic content (Metagenomic Dataset from Tara Oceans). We compared the performance of our integrated workflow against standalone applications of MetaGeneMark/metaProdigal and MetaEuk (Fig. 3). For eukaryotic gene prediction, our workflow yielded a quantity and average length of genes closely comparable to those obtained by using MetaEuk alone (Fig. 3). In contrast, when compared to standalone prokaryotic predictors (MetaGeneMark/metaProdigal), our workflow identified fewer eukaryotic genes, but these were, on average, considerably longer, suggesting less fragmentation. Regarding prokaryotic genes and viral genes, our workflow predicted 14–18% more genes than standalone prokaryotic predictors. However, the average length of these additional prokaryotic/viral genes was 18–27% shorter.

To further assess the workflow’s performance on mixed metagenomes, we analyzed the DeepMicroClass dataset. The results (Fig. S2) demonstrated that our workflow predicted more genes than the standalone methods, although the mean gene length was marginally shorter than that obtained with metaProdigal alone.

## Discussion

The rapid advancement of metagenomic sequencing analysis has spurred the development of specialized tools for gene prediction (Navgire et al., 2022); however, a persistent challenge lies in the comprehensive and accurate annotation of coding sequences across both prokaryotic and eukaryotic domains from mixed microbial community data. Our comparative assessment using representative single-species genomes confirmed distinct performance profiles: while prokaryotic predictors like MetaGeneMark accurately identify prokaryotic genes, they tend to erroneously fragment eukaryotic genes. Conversely, the eukaryotic gene predictor MetaEuk excels at identifying eukaryotic genes with high fidelity and also captures a smaller, yet reliable, subset of prokaryotic coding genes. These observations highlight the inadequacy of standalone tools for robust, cross-domain gene prediction in complex metagenomes.

The primary difficulty in analyzing metagenomes with substantial eukaryotic content is the accurate and efficient prediction of complete coding genes, particularly intron-containing eukaryotic genes, alongside prokaryotic genes. Our study demonstrates that a strategically designed, staged workflow effectively addresses this. By prioritizing the initial identification of eukaryotic genes using MetaEuk, our workflow preserves the integrity of these sequences, thereby avoiding the extensive fragmentation typically observed when prokaryotic predictors are directly applied to eukaryotic DNA. While MetaEuk itself identifies some prokaryotic and viral genes, our integrated approach further enhances the recovery of these genes by approximately 14–18% through a subsequent targeted prediction using MetaGeneMark or metaProdigal on sequences masked by the initial MetaEuk predictions. Importantly, this workflow ensures that the quantity and average length of predicted eukaryotic genes remain comparable to those obtained using MetaEuk alone, while significantly improving the yield and diversity of prokaryotic and viral genes compared to the application of standalone prokaryotic tools. The observation that these additionally predicted prokaryotic/viral genes have a shorter average length warrants further investigation. This could potentially indicate the recovery of partial or incomplete genes from fragmented contigs, or alternatively, complete genes from organisms that inherently possess shorter coding sequences, which might be overlooked by more stringent standalone prokaryotic predictors (Li et al., 2021).

Our validation with the DeepMicroClass dataset further confirms the robustness of the workflow. Compared with standalone MetaEuk or Metagenemark, the integrated approach yielded more genes with comparable annotation completeness, indicating enhanced prediction precision for mixed metagenomic environments. Moreover, while tools such as DeepMicroClass and Whokaryote (Pronk & Medema, 2022) specialize in separating contigs by domain prior to gene prediction, our workflow focuses on gene-level integration across domains after assembly. These approaches are complementary, and future versions of our pipeline could incorporate such classification steps to improve pre-processing efficiency. As sequencing costs continue to decrease and the scale of metagenomic datasets expands, the computational efficiency of each analytical step becomes increasingly critical. Conventional alignment-based masking approaches, such as those employed by tools like RepeatMasker (often designed for repeat identification rather than gene masking), are prohibitively slow for processing large metagenomic datasets after initial gene prediction. Our custom Perl script circumvents this significant bottleneck. By directly parsing gene coordinates from MetaEuk’s output and performing in-memory replacement of coding regions with ‘N’ characters, this script drastically reduces masking time from days or weeks to mere minutes or hours. This efficiency is a cornerstone of our workflow’s practicality and scalability, enabling the rapid and comprehensive retrieval of both eukaryotic and prokaryotic coding sequences.

In conclusion, the integrated workflow presented here offers a pragmatic and efficient solution for the simultaneous prediction of prokaryotic and eukaryotic genes from metagenomic data, particularly from samples rich in eukaryotic content. By leveraging the strengths of domain-specific predictors in a sequential manner, coupled with a highly efficient custom masking procedure, our approach enhances gene recovery while maintaining the integrity of eukaryotic gene structures. The consolidated and more comprehensive gene sets generated by this workflow provide a robust foundation for subsequent downstream functional analyses, such as metabolic pathway reconstruction and phylogenetic studies, thereby facilitating deeper investigations into the functional roles and interactions within complex microbial ecosystems.

## Conclusions

The new workflow effectively enables the rapid and precise retrieval of both prokaryotic and eukaryotic microbial genes from metagenomes.

## Supplemental Information

10.7717/peerj.20769/supp-1 Supplemental Information 1 Prediction results of five archaeal genes

10.7717/peerj.20769/supp-2 Supplemental Information 2 Prediction results of five bacterial genomes

10.7717/peerj.20769/supp-3 Supplemental Information 3 Comparison of the new workflow with standalone MetaEuk and Metagenemark gene prediction pipelines using real data from the DeepMicroClass dataset Comparison of the new workflow with standalone MetaEuk and Metagenemark gene prediction pipelines using real data from the DeepMicroClass dataset. The numbers and average lengths of predicted prokaryotic, eukaryotic, and viral genes were compared. The results show that the proposed workflow predicted fewer total genes than the standalone tools but yielded longer average gene lengths, indicating reduced fragmentation and improved prediction accuracy.

10.7717/peerj.20769/supp-4 Supplemental Information 4 Prediction results of five fungi genomes

10.7717/peerj.20769/supp-5 Supplemental Information 5 Prediction results of five metazoans genomes

10.7717/peerj.20769/supp-6 Supplemental Information 6 Prediction results of five plant genomes

10.7717/peerj.20769/supp-7 Supplemental Information 7 Prediction results of five protozoan genomes MetaGeneMark (predicting prokaryotic gene) and MetaEuk (predicting eukaryotic gene) were used to predict the genes of five representative species of archaea, bacteria, fungi, metazoa, plants and protozoa. To evaluate the reliability of two types of software in predicting prokaryotic and eukaryotic genes from prokaryotic and eukaryotic genomes, respectively.

10.7717/peerj.20769/supp-8 Supplemental Information 8 Genetic information statistics

## Additional Information and Declarations

### Data Availability

The following information was supplied regarding data availability: The data is available at MetaEuk: https://wwwuser.gwdg.de/ compbiol/metaeuk/. The code is available at GitHub and Zenodo: – https://github.com/wzhangahas/PPEMS. – Zhang, W. (2025). Parsing Prokaryotic and Eukaryotic Microbial Genes Simultaneously from Metagenome by a Novel Analysis Workflow. Zenodo. https://doi.org/10.5281/zenodo.18027880.
