# Supplementary figures and images for: A novel analysis workflow for simultaneous parsing prokaryotic and eukaryotic microbial genes from metagenomes
Source: PeerJ. 2026 Feb 11;14:e20769. doi: 10.7717/peerj.20769 (PMC12906264; doi:10.7717/peerj.20769)

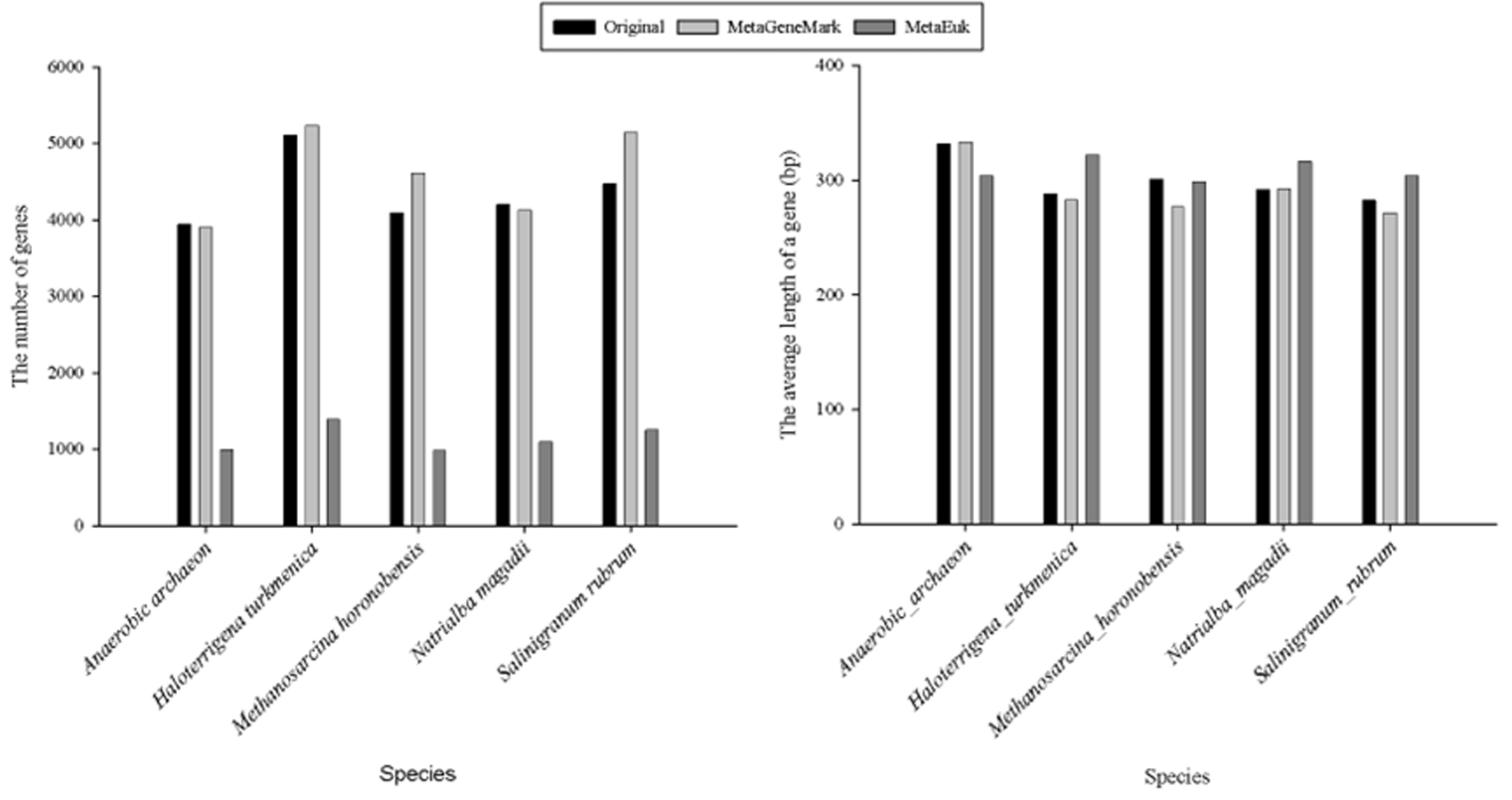

Supplement: Supplemental Information 1 [file peerj-14-20769-s001.png]

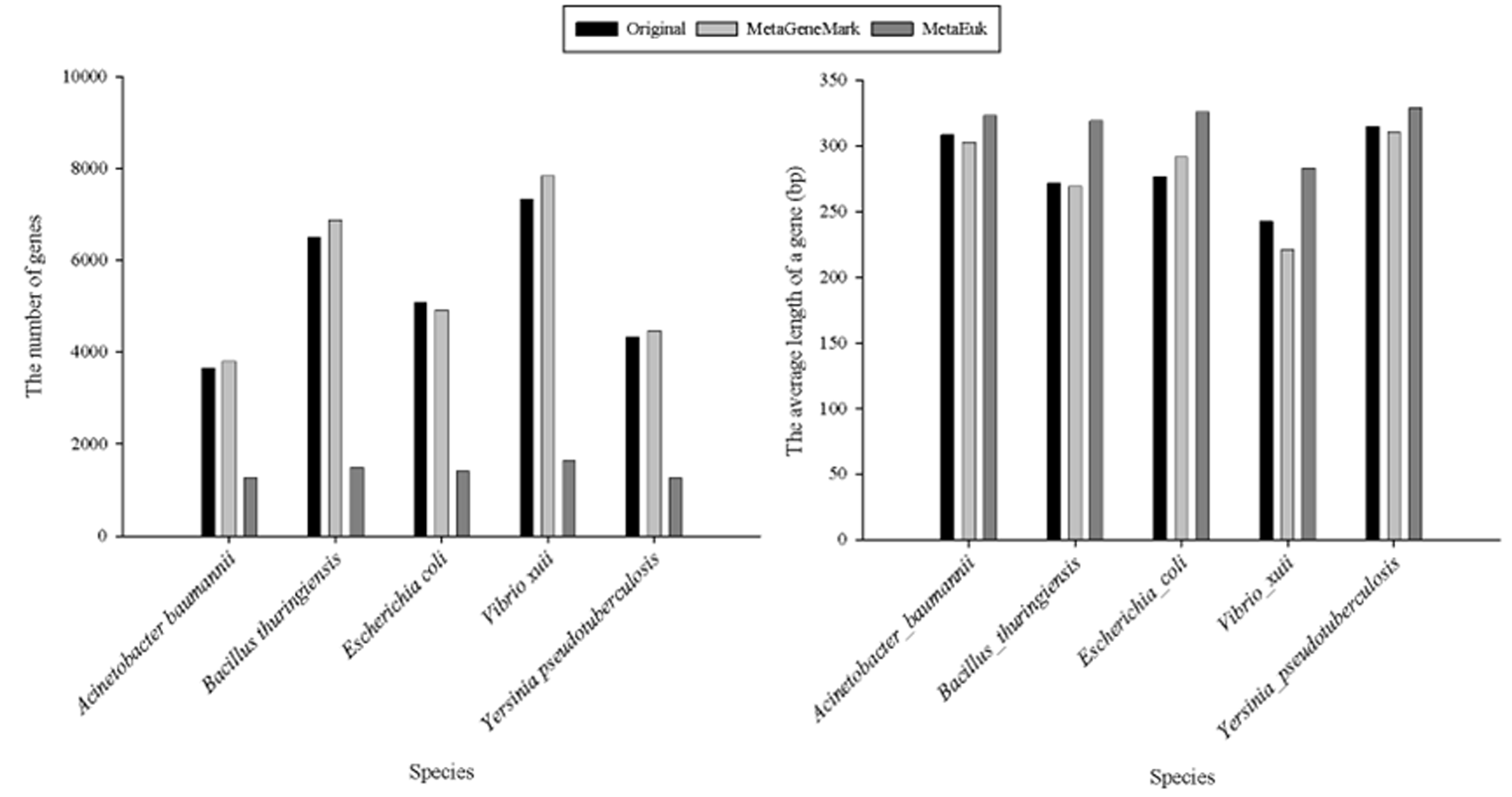

Supplement: Supplemental Information 2 [file peerj-14-20769-s002.png]

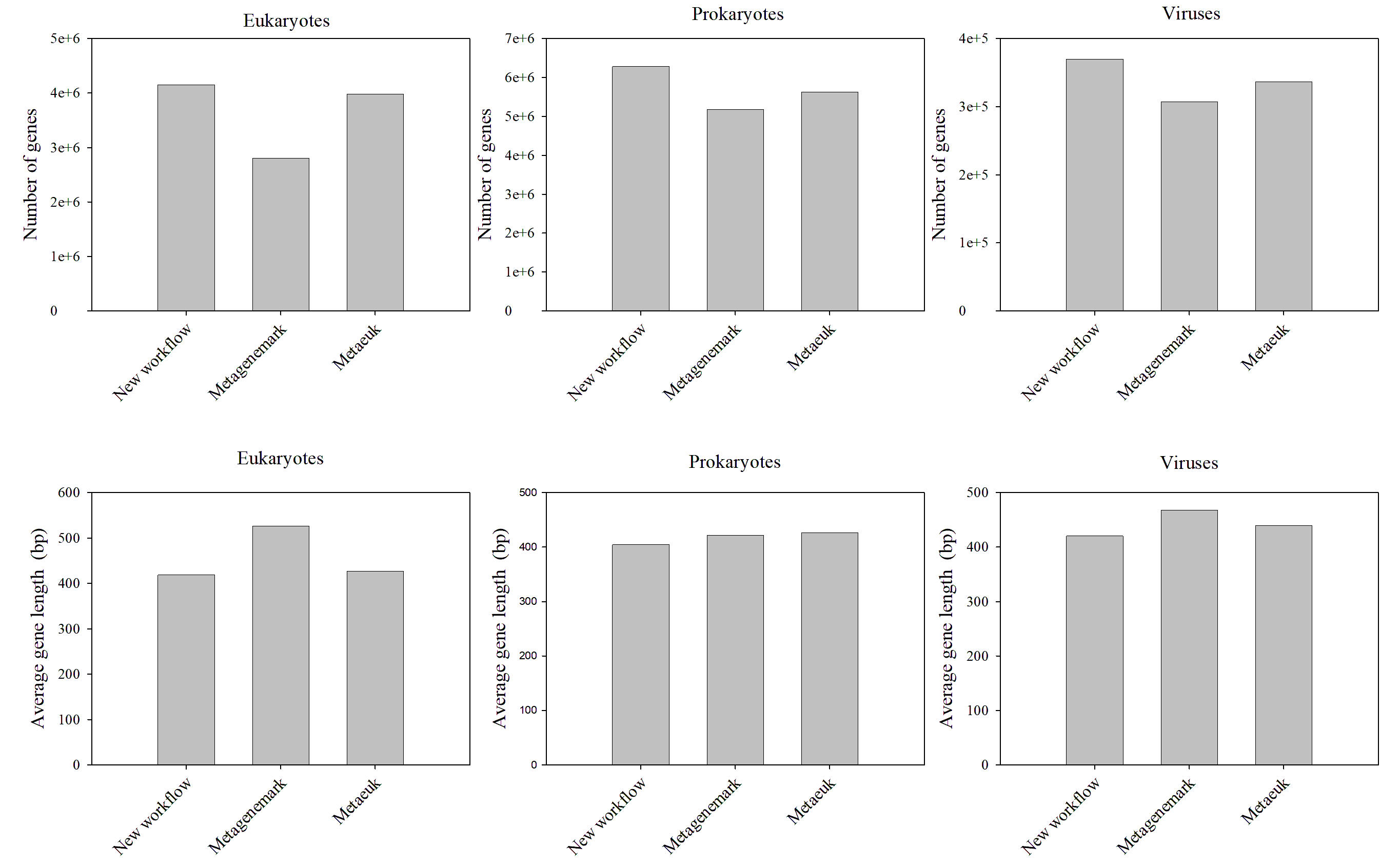

Supplement: Supplemental Information 3 — Comparison of the new workflow with standalone MetaEuk and Metagenemark gene prediction pipelines using real data from the DeepMicroClass dataset. The numbers and average lengths of predicted prokaryotic, eukaryotic, and viral genes were compared. The results show that the proposed workflow predicted fewer total genes than the standalone tools but yielded longer average gene lengths, indicating reduced fragmentation and improved prediction accuracy. [file peerj-14-20769-s003.png]

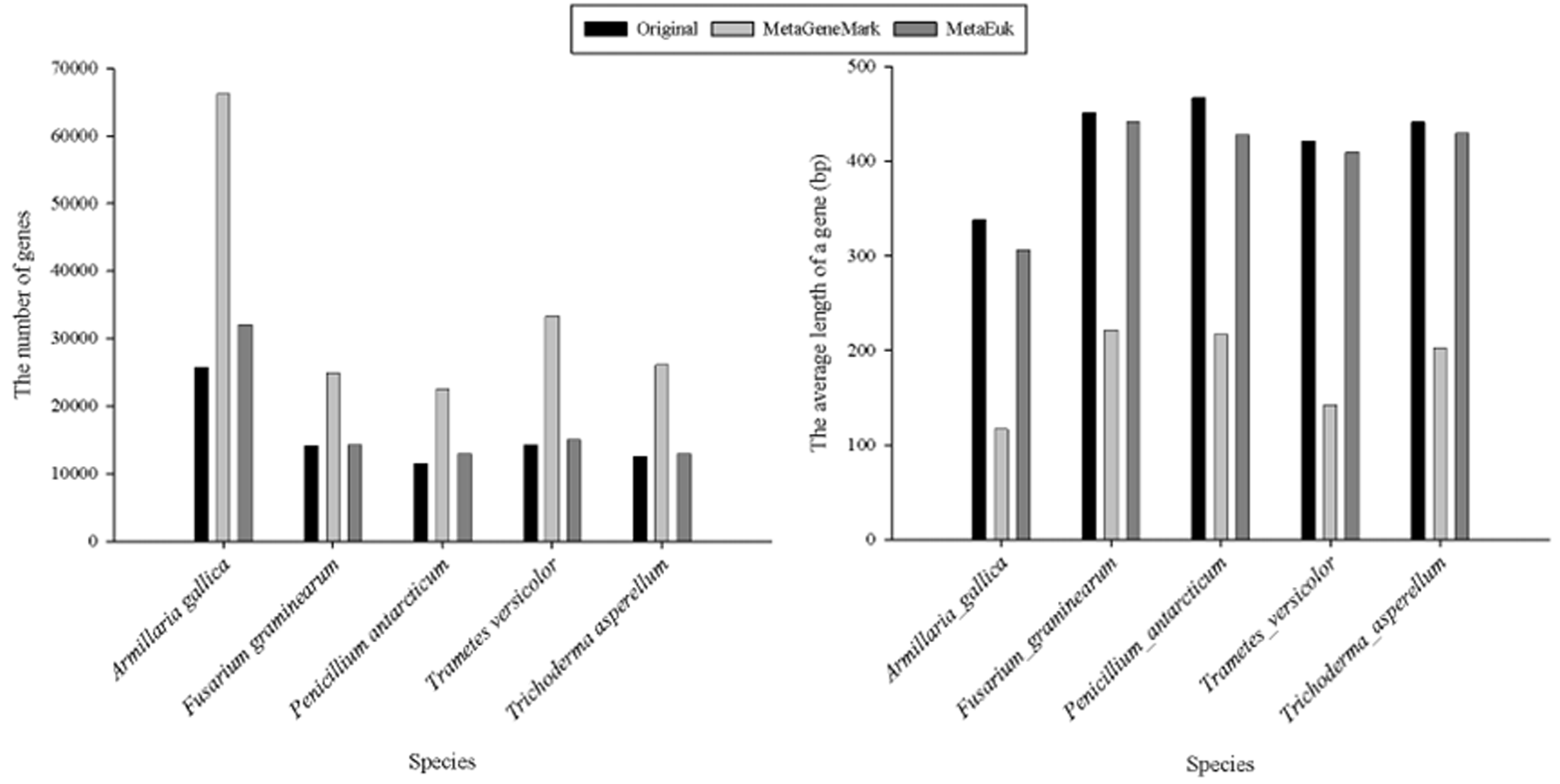

Supplement: Supplemental Information 4 [file peerj-14-20769-s004.png]

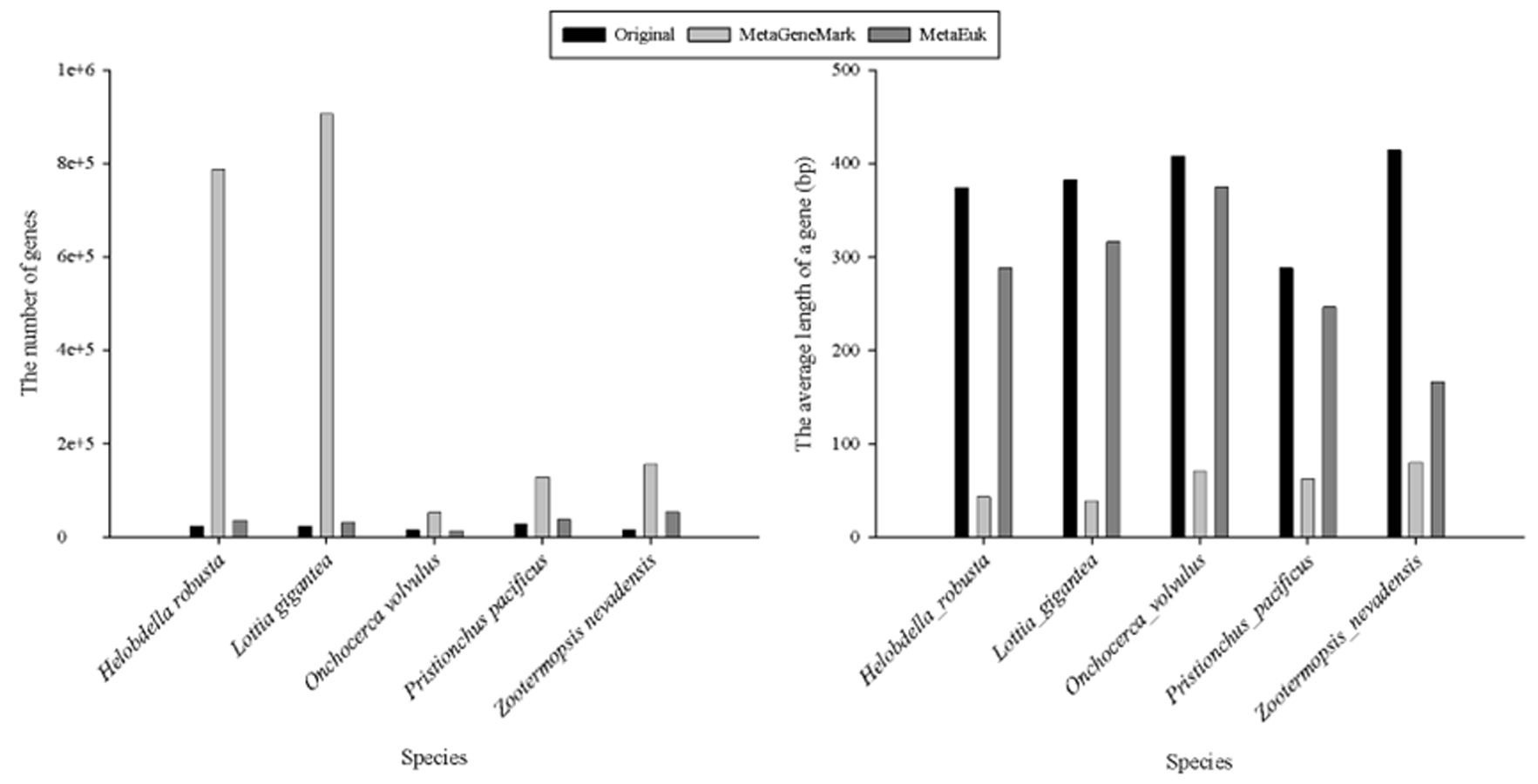

Supplement: Supplemental Information 5 [file peerj-14-20769-s005.png]

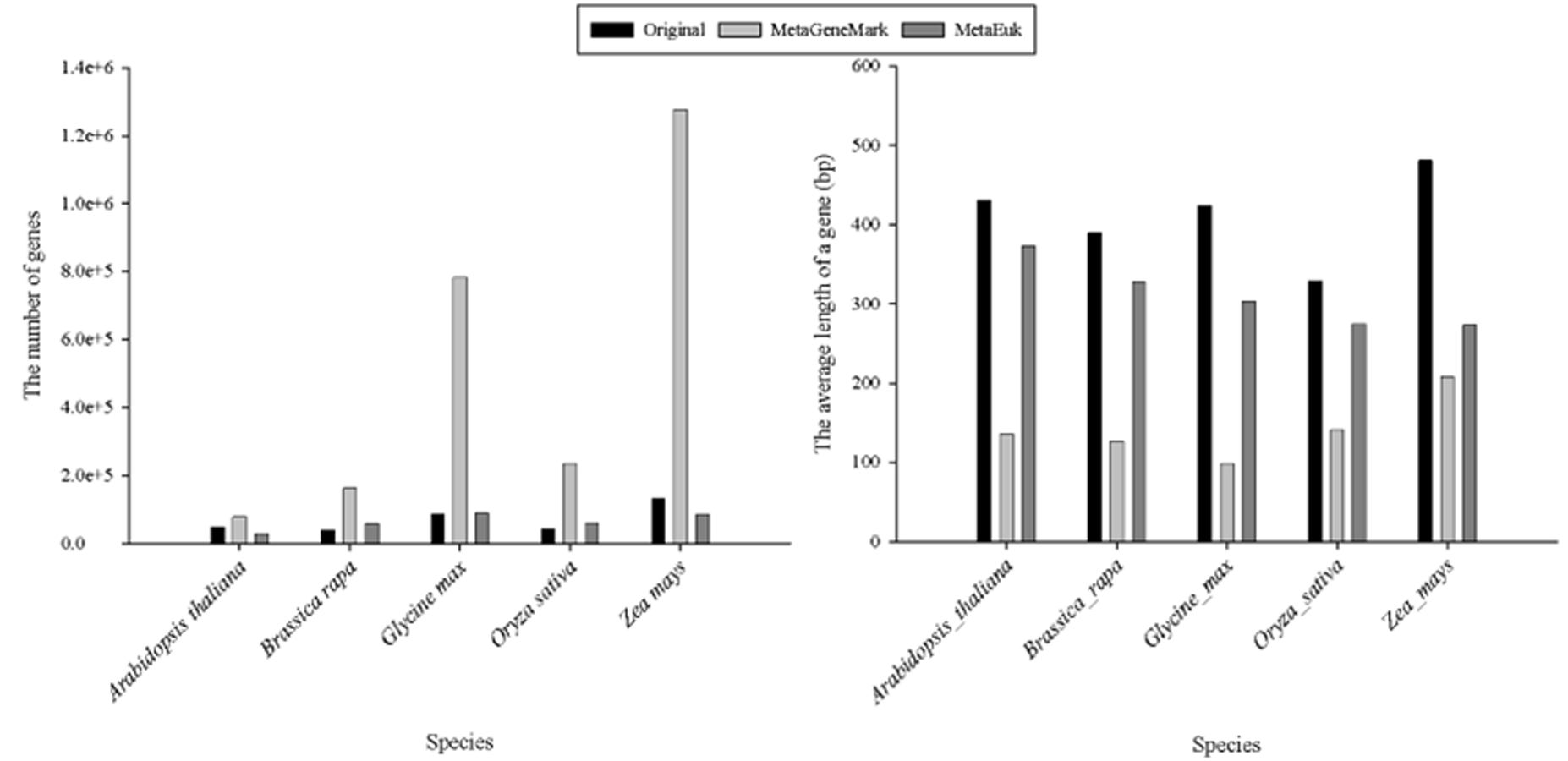

Supplement: Supplemental Information 6 [file peerj-14-20769-s006.png]

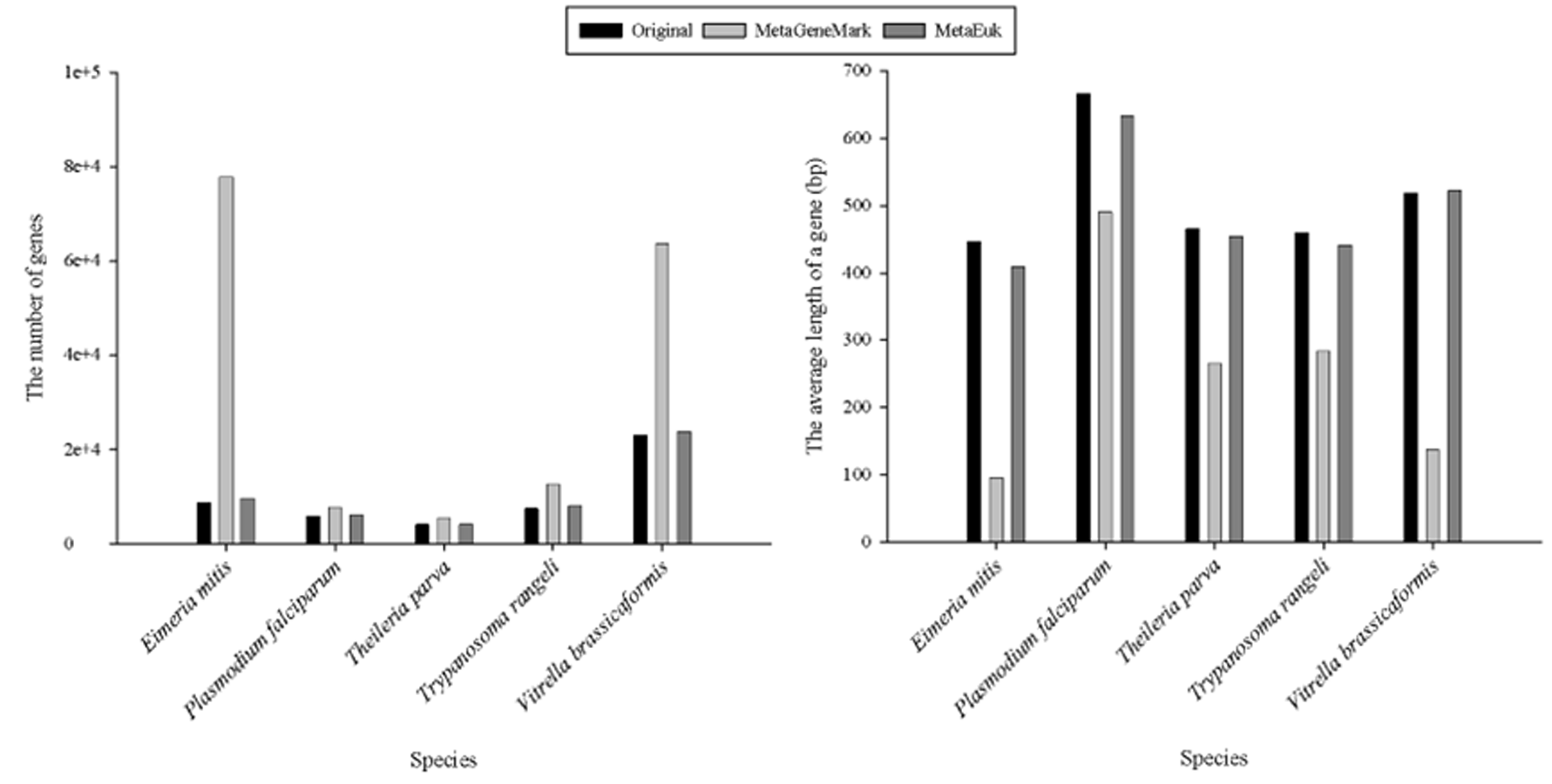

Supplement: Supplemental Information 7 — MetaGeneMark (predicting prokaryotic gene) and MetaEuk (predicting eukaryotic gene) were used to predict the genes of five representative species of archaea, bacteria, fungi, metazoa, plants and protozoa. To evaluate the reliability of two types of software in predicting prokaryotic and eukaryotic genes from prokaryotic and eukaryotic genomes, respectively. [file peerj-14-20769-s007.png]
