# Supplementary material for: A novel analysis workflow for simultaneous parsing prokaryotic and eukaryotic microbial genes from metagenomes
Source: PeerJ. 2026 Feb 11;14:e20769. doi: 10.7717/peerj.20769 (PMC12906264; doi:10.7717/peerj.20769)
Supplement: Supplemental Information 8 [file peerj-14-20769-s008.docx]

Supplementary Table 1. Genetic information statistics

|  | Pipeline | Number of genes | Average gene length (bp) |
| --- | --- | --- | --- |
| Eukaryotes | Metaeuk+Metagenemark | 3515924 | 1325.67 |
|  | Metaeuk+Prodigal | 3387771 | 1392.75 |
|  | MetaGeneMark | 3577830 | 1180.99 |
|  | Prodigal | 3732165 | 1255.48 |
|  |  |  |  |
|  | Pipeline | Number of genes | Average gene length (bp) |
| Prokaryotes | Metaeuk+Metagenemark | 591783 | 912.22 |
|  | Metaeuk+Prodigal | 560112 | 953.33 |
|  | MetaGeneMark | 515441 | 1065.27 |
|  | Prodigal | 512353 | 1153.12 |
|  |  |  |  |
|  | Pipeline | Number of genes | Average gene length (bp) |
| Viruses | Metaeuk+Metagenemark | 214531 | 828.97 |
|  | Metaeuk+Prodigal | 204269 | 897.14 |
|  | MetaGeneMark | 185652 | 973.90 |
|  | Prodigal | 181410 | 1059.39 |
